# Molecular epidemiology of extended-spectrum beta-lactamase-producing Klebsiella pneumoniae bloodstream infections from Aberdeen, Scotland, and their comparison to isolates from England

**DOI:** 10.1099/mgen.0.001413

**Published:** 2025-06-24

**Authors:** Bruno Silvester Lopes, Hisham N. Altayb, Kareem Mahdy, Noha EI Sakka

**Affiliations:** 1 School of Health and Life Sciences, Teesside University, Middlesbrough, UK; 2 National Horizons Centre, Teesside University, Darlington, UK; 3 Department of Biochemistry, Faculty of Science, King Abdulaziz University, Jeddah, Saudi Arabia; 4 Center of Artificial Intelligence in Precision Medicines, King Abdulaziz University, Jeddah, Saudi Arabia; 5 Aberdeen Royal Infirmary, NHS Grampian, Foresterhill, Aberdeen, UK; 6 School of Medicine, Medical Sciences and Nutrition, University of Aberdeen, Aberdeen, UK

**Keywords:** antibiotic resistance, cephalosporins, extended-spectrum *β*-lactamase (ESBL), *Klebsiella pneumoniae*, *mcr*, plasmids, whole-genome sequencing

## Abstract

*Klebsiella* spp., the second leading cause of bacteraemia in the UK, are major nosocomial pathogens. We investigated the epidemiology of extended-spectrum *β*-lactamase (ESBL)-producing *Klebsiella* spp. in Aberdeen, Scotland and England. Twenty-four ESBL producers underwent whole-genome sequencing and typing using MLST. Sequence types were queried in the Bacterial Isolate Genome Sequence Database (BIGSdb) to assess phylogenetic relationships using Parsnp, and resistance/virulence genes were identified by Kleborate. Plasmids were assembled with plasmidSPAdes. All isolates had the *bla*_SHV_, with three positive for *bla*_SHV-12_ ESBL and two positive for *bla*_DHA-1_ ESBL. The *bla*_CTX-M_ was observed in 71% (33% plasmid-borne), *bla*_TEM_ in 42%, with all isolates resistant to ceftazidime, cefotaxime and cefuroxime. Two isolates carried *mcr-9.1* on 2.5 and 203 kb plasmids. MLST identified 21 strains, with ST-13 and ST-200 being the most common. BIGSdb revealed 487 similar strains across 43 clonal groups. ST14 (34%) and ST15 (29%) were predominant multidrug-resistant strains. Nearly all isolates (99%) harboured the *bla*_SHV_ (43% SHV-28, 2% SHV-12 ESBL), and 57% carried a *bla*_CTX-M_. Carbapenemases were detected in 61% of isolates, and 59% had *gyr*A/*par*C mutations, exclusively linked to strains from England. ST432 strains from humans (resistant) and cattle (sensitive) were closely related. We also observed ST14 and ST15 (CC15) as high-risk clones thought to have evolved independently that are globally prevalent. The coexistence of *mcr-9.1* and plasmid ESBL *bla*_CTX-M_ in *Klebsiella* spp. threatens the efficacy of critically important antibiotics. The close relatedness of human and cattle ST432 strains suggests potential zoonotic transmission, emphasizing the role of livestock as a reservoir for antimicrobial-resistant pathogens and the need for genomic epidemiology studies to elucidate transmission pathways and interspecies exchange. A One Health approach integrating human, animal and environmental surveillance would enhance our understanding of the molecular dynamics and evolutionary trajectory of *Klebsiella pneumoniae*, reinforcing the need for targeted interventions to limit the spread of antimicrobial resistance.

**Impact Statement**

*Klebsiella pneumoniae* is designated by the World Health Organization as a critical-priority pathogen and is a leading cause of bacterial bloodstream infections worldwide. Understanding its molecular epidemiology, antimicrobial resistance and virulence mechanisms is essential for understanding its genetic diversity and the evolution of emerging clones. However, resource limitations often pose a barrier in restricting our ability to perform whole-genome sequencing (WGS) on every isolate. To address this, we analysed 719 isolates collected from Aberdeen Royal Infirmary, Scotland, selecting 24 extended-spectrum beta-lactamase-positive samples for WGS. These were then compared with isolates from England deposited in the Bacterial Isolate Genome Sequence Database public database to gain insights into resistance patterns and genetic variations across regions. Our findings revealed a notably higher rate of resistance, particularly to carbapenems, amongst strains from England compared to Scotland. Additionally, sequence types ST14 and ST15 were identified as high-risk clones, underscoring their endemic presence across the UK. This study highlights the resistance and virulence mechanisms in *K. pneumoniae* using microbial genomics and emphasizes the need for enhanced surveillance, rigorous infection control and novel therapeutics to mitigate the growing threat of antibiotic-resistant infections in healthcare, helping in improving public health.

## Data Summary

This Whole Genome Shotgun project has been deposited at DDBJ/ENA/GenBank under BioProject ID PRJNA1181535 with accession numbers JBJABU000000000–JBJACR000000000. The isolates are also listed in the Supplementary Data files with BIGS database accession IDs which are linked to the accession numbers. The BIGS *Klebsiella* database is accessible via https://bigsdb.pasteur.fr/cgibin/bigsdb/bigsdb.pl?db=pubmlst_klebsiella_isolates. All other supporting data have been provided within the article or in the supplementary data files.

## Introduction

*Klebsiella pneumoniae* is a gram-negative, encapsulated, non-motile bacterium found in the environment which is associated with pneumonia, bloodstream infections (BSIs), meningitis and urinary tract infections [1]. It has also been associated with poor outcomes in older patients with alcohol use disorder or diabetes mellitus. According to the recent taxonomic classification, there are at least seven phylogroups of *K. pneumoniae* (Kp1–Kp7) which contribute to the *K. pneumoniae* species complex (KpSC). The KpSC consists of five species, which include *K. pneumoniae sensu stricto* (Kp1), which is the most clinically relevant group that includes hypervirulent and multidrug-resistant strains; *Klebsiella quasipneumoniae*, which has two subspecies – *quasipneumoniae* (Kp2) and subsp. *similipneumoniae* (Kp4); and *Klebsiella variicola*, which was initially thought to be an environmental or plant-associated species with two subspecies – *variicola* (Kp3) and subsp. *tropica* (Kp5) – and was previously misnamed as *Klebsiella tropicalensis*. Other than that, *Klebsiella quasivariicola* (Kp6) has limited clinical data, and *Klebsiella africana* (Kp7), previously misnamed as *Klebsiella africanensis*, also forms part of KpSC [2,3].

*Klebsiella* spp. usually colonize the gastrointestinal tract and oropharynx mucosal surfaces in humans where they do not normally cause disease, but infections can be acquired endogenously from the patient’s own gut flora or exogenously from the healthcare environment. Patient-to-patient transmission can occur through contaminated hands of hospital personnel or less commonly by contamination of surfaces in the hospital environment. Immune-compromised patients and young children, including infants, are at the most risk of infections which can be associated with the use of invasive devices or medical procedures. *Klebsiella* spp. have overtaken methicillin-resistant *Staphylococcus aureus* in the UK, with an increase in the percentage of hospital-onset bacteraemia cases which were also linked with the severe acute respiratory syndrome coronavirus-2 [4]. *K. pneumoniae* currently accounts for the majority of BSIs in the UK with around 8% of hospital-acquired infection cases and is significantly associated with morbidity and mortality [5,6]. There continues to be an increase in BSIs due to *Klebsiella* spp. despite it being a public health priority, and it is not clear how the ambitious targets to reduce infections set out by the UK government can be achieved. The mortality rate of BSI due to *K. pneumoniae* in most studies has ranged from 32.6% to 56.1% but was higher (up to 79%) in carbapenem-resistant *K. pneumoniae*, leading to higher mortality [7]

The recently released 2024 report on the World Health Organization’s (WHO) Bacterial Priority Pathogens lists third-generation cephalosporin-resistant and carbapenem-resistant *K. pneumoniae* as a critical priority pathogen that needs urgent attention [8]. It also highlights that the new list has almost remained unchanged since 2017 when it was first published. Multidrug-resistant *K. pneumoniae* (MDR-KP) is a major threat, especially in hospitals, where it causes infections like pneumonia and sepsis. Its high resistance to antibiotics makes treatment difficult, highlighting the need for better monitoring, new treatments and stronger infection control. Our study supports WHO’s concerns by offering key insights into the spread of resistance and its implications to One Health, with global efforts being needed to tackle this growing health crisis. *K. pneumoniae* is well known for its ability to acquire genetic elements through horizontal gene transfer and is broadly classified as MDR-KP and hypervirulent *K. pneumoniae* (HvKP). MDR-KP produces extended-spectrum *β*-lactamases (ESBLs) like TEM, SHV variants and CTX-M enzymes, as well as carbapenemases such as KPC, NDM, VIM and OXA-48-like enzymes, which contribute to treatment failures in various infections [9,10]. It is also important to note that changes in therapeutic options and perhaps the use of different antimicrobials may be responsible for the epidemiological variation in resistant strains of *Klebsiella* spp. which commonly possessed the TEM and SHV variants that emerged in the 1980s and CTX-M-15 during the 2000s [9]. This marks a clear shift in the development of resistance which reflects the organism’s capability to evolve using mechanisms like gene transfer, possibly between different epidemic clone types [11]. The HvKP carries integrative conjugative elements (ICEs) and plasmids encoding virulence factors such as the colibactin toxin gene *clb*; siderophore genes *iro*, *iuc* and *ybt*; and regulators for mucoid phenotype *rmpA*/*rmpA2*, which enable it to cause community-acquired infections even in healthy individuals [7]. Clones such as ST11, ST15, ST101, ST147 and ST258 are widely circulating for more than two decades, with ST307, ST383, ST392, ST405, ST512 and multidrug-resistant ST2096 emerging recently in different parts of the world [11,14].

There have been very limited studies on understanding the molecular epidemiology of *Klebsiella* spp. in Scotland, despite it being the second most common cause of bacteraemia after *Escherichia coli* [15]. The incidence of both *K. pneumoniae* and *Klebsiella oxytoca* bacteraemia over the last 5 years in Scotland has remained stable from 2015 to 2019 equating to 14.2 and 3.9 per 100,000 population, respectively. This is comparable to an incidence of 12.5 and 2.8 per 100,000 population which has been observed in England, Wales and Northern Ireland. This study aimed to identify cases of ESBL-producing *K. pneumoniae* complex BSIs in Aberdeen, Scotland, and to investigate their molecular epidemiology using whole-genome sequencing. Additionally, we compared our isolates with those from other regions in the UK to gain insights into strain diversity and the similarities or differences in antibiotic resistance mechanisms.

## Methods

### Bacterial isolates and genotyping

Patients with suspected bacteraemia had their blood cultures incubated in BD BACTEC bottles, and positive samples were grown on MacConkey agar which were initially identified by MALDI-TOF. A total of 719 isolates of *K. pneumoniae* spp. complex were collected from patients in Aberdeen Royal Infirmary between 2017 and 2022, with a non-duplicate subset of 24 ESBL-positive isolates from 2019 to 2022 that were whole-genome sequenced. A single pure culture colony was streaked onto nutrient agar and incubated at 37 °C for 24 h. Half to two-thirds of plate growth was harvested and resuspended in 5 ml of PBS. The culture was centrifuged to reach an OD of 10 in 1 ml. The supernatant was discarded, and a maximum of 4×10^9^–6×10^9^ cells (wet pellet weight of 30–50 mg) was resuspended in the inactivation buffer provided by MicrobesNG. We used the MicrobesNG Hybrid service that offers both long- and short-read sequencing and combines data from the latest R10.4.1 chemistry from Oxford Nanopore Technologies with 2×250 bp Illumina reads to obtain the highest-quality assemblies for our bacterial genomes. The *de novo* hybrid assemblies of Nanopore and Illumina reads were assembled using the Unicycler pipeline v.0.4.0. The genomes were uploaded to the Bacterial Isolate Genome Sequence Database (BIGSdb) and typed using the MLST and strict (synteny-controlled) core genome scheme (scgMLST629_S) [16,17].

### Antimicrobial susceptibility testing

We tested our isolates for antimicrobial susceptibility using the agar double dilution method (MIC) using Iso-Sensitest agar (Oxoid, UK). The antibiotics tested belonged to the penicillin class: amoxicillin, ampicillin, piperacillin/tazobactam, amoxicillin/clavulanic acid and temocillin; cephalosporins: cefoxitin, cefuroxime, cefotaxime and ceftazidime; aminoglycosides: amikacin, gentamicin (GEN) and tobramycin (TOB); fluoroquinolones: ciprofloxacin; sulphonamides: trimethoprim/sulfamethoxazole and trimethoprim; glycylcycline: tigecycline; and carbapenems: ertapenem and meropenem. The MIC for isolates positive for colistin resistance genes was performed using the broth micro-dilution method at Newcastle Laboratories (Newcastle, UK). The results were interpreted according to the EUCAST guidelines [18].

### Antimicrobial and virulence gene determinants

The 24 genomes were annotated using RAST [19]. Antibiotic resistance genes were screened in the RAST database, along with using ResFinder, and plasmids were detected using PlasmidFinder. Kleborate (v2.0.0) functionality built in BIGSdb was used for detecting ICE*Kp*-associated virulence loci [yersiniabactin (*ybt*), colibactin (*clb*) and salmochelin (*iro*)] and virulence plasmid-associated loci [salmochelin (*iro*), aerobactin (*iuc*) and hypermucoidy (*rmpA* and *rmpA2*)]. Antimicrobial resistance (AMR) determinants were determined, along with intrinsic *β*-lactamases and K (capsule) and O antigen (LPS) serotype prediction, *wzi* alleles and Kaptive [16,19, 20].

### AMR and phylogeny of *Klebsiella* across England and Scotland

The BIGSdb (https://bigsdb.pasteur.fr/klebsiella/, last accessed first June 2024) was screened for the presence of *K. pneumoniae* spp. complex genomes resembling the ones observed in our study [16]. Data related to strains from England, UK, were extracted and compared to our whole-genome-sequenced isolates. The whole-genome phylogenetic trees of *K. pneumoniae* isolates were constructed using Parsnp and Harvest suite using the standard setting with *K. pneumoniae* MGH78578 as the reference strain [16,21, 22]. The genomic phylogenetic tree was visualized using the Interactive Tree of Life (iTOL) platform. Metadata for the tree visualization was generated using the itol.toolkit R package, facilitating the annotation and customization of the tree [23]. These isolates were further analysed to provide an overview of the phylogenetic relationships and molecular epidemiology in the context of cases from England. Similarly, a phylogenetic tree was constructed for all Scottish isolates from the BIGSdb, along with our 24 isolates, to understand the relationships amongst the Scottish strains. AMR and virulence genes in these isolates were screened using the BIGSdb Kleborate (v2.0.0) tool [20]. A detailed analysis was conducted on genes conferring resistance to penicillin, third-generation cephalosporins, carbapenems, colistin, trimethoprim-sulphonamides, chloramphenicol, macrolides, tetracyclines and mutations associated with the quinolone resistance-determining regions of *gyr*A and *par*C.

### Plasmid assembly and screening

Plasmids from our 24 isolates were assembled from raw sequence data using plasmidSPAdes, an assembler specifically designed for short-read sequencing data [24]. The assembled plasmid DNA was then analysed using Kleborate [20]. To construct a phylogenetic tree based on the presence and absence of variants (PAVs) (binary data), we employed Panseq, utilizing the following parameters: novelRegionFinderMode set to no_duplicates, sequences fragmented into smaller sections of 1,000 bp and a per cent identity cutoff of 99% which was applied [25]. The binary.phylip files which contain PAVs were used as input for phylogenetic tree construction using RAxML, where the MULTIGAMMA model was employed for the analysis, and the number 1000 was set for bootstrap replicates [26]. The plasmid phylogenetic trees were visualized using the iTOL platform, with the itol.toolkit R package enabling tree annotation and customization, including the integration of metadata [23].

## Results

### *Klebsiella* bacteraemia in England and Aberdeen, Scotland

The annual counts and rates of *Klebsiella* spp. bacteraemia from 2017 to 2022 by NHS acute trust and sub-integrated care board location are available from the UKHSA website (https://www.gov.uk/government/statistics/klebsiella-species-bacteraemia-annual-data, last accessed 31 July 2024). This includes 137 trusts in England from where data was collected along with data from Aberdeen Royal Infirmary (Table 1). Between 2017/2018 and 2022/2023, 27 NHS trusts reported a decrease of 1%–50% in *Klebsiella* spp. bacteraemia cases, five showed no change (0%) and four could not be evaluated due to small sample sizes. Notably, 79 trusts experienced an increase of 1%–48%, whilst 22 saw a major rise from 51% to 482%, contributing to the growing burden of *Klebsiella* spp. bacteraemia in the UK. Overall, there was a 21% increase in cases across England and a 29% increase recorded in Aberdeen Royal Infirmary in Aberdeen, Scotland (Table 1).

**Table 1.** Number of *Klebsiella* spp. BSIs across England and Aberdeen, Scotland. Red box highlights an increase of more than 50%, green box highlights a decrease of less than 50% and yellow box highlights no change during a 5-year period

Organization	Code	2017/2018	2018/2019	2019/2020	2020/2021	2021/2022	2022/2023	% increase/decrease
Airedale	RCF	28	13	27	22	31	31	11%
Alder Hey Children’s	RBS	10	18	17	17	14	20	100%
Ashford and St Peter’s Hospitals	RTK	53	59	48	54	51	45	−15%
Barking, Havering and Redbridge University Hospitals	RF4	134	145	118	165	164	169	26%
Barnsley Hospital	RFF	49	28	48	43	49	48	−2%
Barts Health	R1H	246	254	233	309	274	271	10%
Bedfordshire Hospitals NHS Foundation Trust	RC9	80	80	85	82	62	89	11%
Birmingham Women’s and Children’s	RQ3	10	17	17	16	19	27	170%
Blackpool Teaching Hospitals	RXL	72	69	74	70	84	98	36%
Bolton NHS	RMC	54	61	68	43	39	58	7%
Bradford Teaching Hospitals	RAE	46	73	59	61	70	53	15%
Buckinghamshire Healthcare	RXQ	61	56	65	74	62	77	26%
Calderdale and Huddersfield	RWY	59	64	78	67	88	84	42%
Cambridge University Hospitals	RGT	51	96	122	133	141	144	182%
Chelsea and Westminster Hospital	RQM	67	79	81	88	102	101	51%
Chesterfield Royal Hospital	RFS	62	71	62	51	52	38	−39%
Countess of Chester Hospital	RJR	60	54	57	39	63	69	15%
County Durham and Darlington	RXP	89	102	91	95	101	91	2%
Croydon Health Services	RJ6	68	52	64	77	65	65	−4%
Dartford and Gravesham	RN7	39	48	64	55	52	48	23%
Doncaster and Bassetlaw Hospitals	RP5	86	93	99	87	94	91	6%
Dorset County Hospital	RBD	27	42	58	45	50	34	26%
East and North Hertfordshire	RWH	57	76	74	57	69	62	9%
East Cheshire	RJN	19	30	35	14	23	35	84%
East Kent Hospitals University	RVV	183	165	174	141	128	153	−16%
East Lancashire Hospitals	RXR	84	87	83	86	95	76	−10%
East Suffolk and North Essex	RDE	112	122	149	137	135	129	15%
East Sussex Healthcare	RXC	89	92	88	71	102	95	7%
Epsom and St Helier University Hospitals	RVR	81	70	74	87	78	81	0%
Frimley Health	RDU	142	139	154	178	161	201	42%
Gateshead Health	RR7	56	55	58	50	56	71	27%
George Eliot Hospital	RLT	36	29	33	40	25	32	−11%
Gloucestershire Hospitals	RTE	76	85	66	50	49	49	−36%
Great Ormond Street Hospital for Children	RP4	19	19	28	14	16	35	84%
Great Western Hospitals	RN3	55	55	47	53	55	48	−13%
Guy’s and St. Thomas'	RJ1	106	112	136	157	134	139	31%
Hampshire Hospitals	RN5	55	36	82	68	61	69	25%
Harrogate and District	RCD	31	28	31	33	27	40	29%
Homerton University Hospital	RQX	34	32	36	53	39	39	15%
Hull University Teaching Hospitals NHS Trust	RWA	87	106	119	86	121	120	38%
Imperial College Healthcare	RYJ	109	109	118	97	129	116	6%
Isle of Wight	R1F	39	32	29	31	23	40	3%
James Paget University Hospitals	RGP	68	56	63	69	69	59	−13%
Kettering General Hospital	RNQ	45	64	69	64	72	78	73%
King’s College Hospital	RJZ	166	193	195	223	212	228	37%
Kingston Hospital	RAX	35	43	47	46	32	49	40%
Lancashire Teaching Hospitals	RXN	70	71	76	57	75	71	1%
Leeds Teaching Hospitals	RR8	128	207	170	179	188	246	92%
Lewisham and Greenwich	RJ2	98	88	109	100	106	124	27%
Liverpool Heart and Chest Hospital	RBQ	5	2	6	1	3	6	20%
Liverpool University Hospitals NHS Foundation Trust	REM	153	180	192	167	178	177	16%
Liverpool Women’s	REP	4	1	2	2	2	3	−25%
London North West University Healthcare	R1K	114	117	148	165	170	161	41%
Maidstone and Tunbridge Wells	RWF	83	99	73	84	86	79	−5%
Manchester University	R0A	183	228	212	236	233	225	23%
Medway	RPA	72	63	82	69	77	82	14%
Mid and South Essex NHS Foundation	RAJ	182	209	252	198	189	256	41%
Mid Cheshire Hospitals	RBT	29	46	36	42	45	56	93%
Mid Yorkshire Hospitals	RXF	82	85	82	105	107	97	18%
Milton Keynes University Hospital	RD8	31	49	40	27	47	58	87%
Moorfields Eye Hospital	RP6	0	0	0	0	0	0	nd
Norfolk and Norwich University Hospitals	RM1	85	69	83	107	115	75	−12%
North Bristol	RVJ	59	74	79	74	75	72	22%
North Cumbria Integrated Care NHS Foundation Trust	RNN	56	62	42	52	78	69	23%
North Middlesex University Hospital	RAP	48	70	78	59	75	84	75%
North Tees and Hartlepool	RVW	71	60	59	50	60	71	0%
North West Anglia	RGN	83	96	65	99	133	123	48%
Northampton General Hospital	RNS	66	54	71	63	47	50	−24%
Northern Care Alliance NHS Foundation Trust	RM3	111	131	143	165	144	158	42%
Northern Lincolnshire and Goole	RJL	59	72	55	62	72	58	−2%
Northumbria Healthcare	RTF	87	97	114	132	79	119	37%
Nottingham University Hospitals	RX1	237	216	243	228	238	239	1%
Oxford University Hospitals	RTH	123	151	176	164	182	167	36%
Portsmouth Hospitals	RHU	84	93	95	93	93	122	45%
Queen Victoria Hospital	RPC	0	0	0	0	0	0	nd
Royal Berkshire	RHW	77	79	90	94	107	88	14%
Royal Cornwall Hospitals	REF	74	103	81	69	92	79	7%
Royal Devon University Healthcare NHS Foundation Trust	RH8	81	106	97	125	120	119	47%
Royal Free London	RAL	106	132	124	143	137	150	42%
Royal National Orthopaedic Hospital	RAN	1	3	1	5	1	0	nd
Royal Papworth Hospital	RGM	7	13	13	28	13	15	114%
Royal Surrey County Hospital	RA2	50	54	69	63	71	94	88%
Royal United Hospitals Bath	RD1	91	78	109	88	75	75	−18%
Salisbury	RNZ	23	23	27	23	26	21	−9%
Sandwell and West Birmingham Hospitals	RXK	48	50	48	47	46	63	31%
Sheffield Children’s	RCU	14	9	6	18	2	11	−21%
Sheffield Teaching Hospitals	RHQ	177	196	174	169	193	224	27%
Sherwood Forest Hospitals	RK5	49	64	59	54	90	62	27%
Shrewsbury and Telford Hospital	RXW	62	87	81	99	70	73	18%
Somerset NHS Foundation Trust	RH5	57	73	79	91	74	96	68%
South Tees Hospitals	RTR	131	134	117	114	110	130	−1%
South Tyneside and Sunderland	R0B	110	114	104	100	120	106	−4%
South Warwickshire	RJC	35	50	33	47	35	51	46%
Southport and Ormskirk Hospital	RVY	49	47	51	47	40	53	8%
St. George’s University Hospitals	RJ7	69	61	88	120	93	120	74%
St. Helens and Knowsley Hospitals	RBN	56	67	67	62	59	65	16%
Stockport NHS	RWJ	67	50	57	53	54	51	−24%
Surrey and Sussex Healthcare	RTP	85	118	106	99	122	102	20%
Tameside Hospital	RMP	21	44	49	50	36	43	105%
The Christie Hospital	RBV	28	24	22	24	38	28	0%
The Clatterbridge Cancer Centre	REN	15	7	7	5	16	28	87%
The Dudley Group	RNA	53	77	55	67	78	56	6%
The Hillingdon Hospitals	RAS	47	32	57	36	45	60	28%
The Newcastle upon Tyne Hospitals	RTD	157	173	208	192	219	203	29%
The Princess Alexandra Hospital	RQW	33	43	42	41	35	52	58%
The Queen Elizabeth Hospital King’s Lynn	RCX	45	55	47	51	70	51	13%
The Robert Jones and Agnes Hunt Orthopaedic Hospital	RL1	0	1	3	1	2	3	nd
The Rotherham	RFR	11	43	49	41	54	64	482%
The Royal Marsden	RPY	23	32	49	45	38	42	83%
The Royal Orthopaedic Hospital	RRJ	1	0	1	0	1	1	0%
The Royal Wolverhampton	RL4	63	79	58	64	72	70	11%
The Walton Centre	RET	5	2	4	6	5	5	0%
Torbay and South Devon	RA9	62	53	62	47	62	42	−32%
United Lincolnshire Hospitals	RWD	83	87	94	109	105	114	37%
University College London Hospitals	RRV	111	129	125	127	94	118	6%
University Hospital Southampton	RHM	79	120	111	112	141	117	48%
University Hospitals Birmingham	RRK	234	249	243	305	304	284	21%
University Hospitals Bristol and Weston NHS Foundation Trust	RA7	100	122	100	114	108	102	2%
University Hospitals Coventry and Warwickshire	RKB	93	79	105	88	102	84	−10%
University Hospitals Dorset	R0D	132	145	142	115	140	113	−14%
University Hospitals of Derby and Burton	RTG	125	140	119	136	138	129	3%
University Hospitals of Leicester	RWE	147	135	181	190	161	162	10%
University Hospitals of Morecambe Bay	RTX	50	70	56	55	60	69	38%
University Hospitals of North Midlands	RJE	142	137	129	158	140	170	20%
University Hospitals Plymouth	RK9	78	82	97	98	101	84	8%
University Hospitals Sussex NHS Foundation Trust	RYR	136	154	165	146	153	170	25%
Walsall Healthcare	RBK	24	34	40	40	54	38	58%
Warrington and Halton Hospitals	RWW	43	44	37	43	57	62	44%
West Hertfordshire Hospitals	RWG	73	92	62	76	72	96	32%
West Suffolk NHS	RGR	33	27	44	31	19	48	45%
Whittington Health	RKE	35	38	33	43	41	41	17%
Wirral University Teaching Hospital	RBL	53	66	62	45	65	68	28%
Worcestershire Acute Hospitals	RWP	74	94	96	90	84	87	18%
Wrightington, Wigan and Leigh	RRF	40	23	32	35	35	20	−50%
Wye Valley	RLQ	16	22	28	35	26	31	94%
Yeovil District Hospital	RA4	42	45	33	48	36	37	−12%
York Teaching Hospital	RCB	142	115	140	135	146	140	−1%
								
Total	138	9,920	10,826	11,192	11,284	11,572	11,970	21%
								
Aberdeen Royal Infirmary	ARI	114	102	118	104	134	147	29%

### Bacterial isolates and genotyping

In our study, a total of 719 *K. pneumoniae* spp. complex isolates were collected from bacteraemia patients at Aberdeen Royal Infirmary between 2017 and 2022. These consisted of *K. pneumoniae* (*n*=541) followed by *K. oxytoca* (*n*=136), *Raoultella planticola* (*n*=13), *Raoultella ornithinolytica* (*n*=12), *Klebsiella aerogenes* (*n*=9), *Klebsiella variicola* (*n*=4), *Klebsiella ozaenae* (*n*=1), two unknown *Klebsiella* spp. and one unknown *Raoultella* sp. Twenty-four *Klebsiella* spp. isolates (16 males and 8 females; age range: 40–83) identified by the disc diffusion test for ESBL production were chosen for whole-genome sequencing to understand their molecular epidemiology and association with other strain prevalence across England, UK. These were identified as *K. variicola* subsp. *variicola* (*n*=1), *K. pneumoniae* (*n*=22) and *K. quasipneumoniae* subsp. *similipneumoniae* (*n*=1) and were confirmed with growth in BD BACTEC bottles and on MacConkey agar with initial identification by MALDI-TOF.

### Antimicrobial susceptibility testing

The susceptibilities to major antimicrobial classes are highlighted in Table S1, available in the online Supplementary Material. All except one that was intermediate were resistant to third-generation cephalosporin ceftazidime. All isolates except three were resistant to the second-generation cephalosporin cefuroxime, and two were resistant to cefoxitin. All isolates were resistant to ampicillin and amoxicillin. Additionally, 19 were resistant to co-amoxiclav, 8 were resistant to GEN, 15 showed resistance or intermediate resistance to ciprofloxacin and 20 were resistant to trimethoprim. All isolates were sensitive to temocillin and meropenem, and a large proportion were sensitive or had intermediate resistance to amikacin. Colistin susceptibility was tested in two isolates positive for *mcr9.1*, and these were sensitive with an MIC of 0.5 mg l^−1^ as determined by the microbroth dilution method.

### Antibiotic resistance and virulence genes

All 24 isolates were whole-genome sequenced, and the genome assemblies were deposited in BIGSdbe (https://bigsdb.pasteur.fr/klebsiella/ last accessed 19 July 2023). The sequence type of isolates, scgMLST629_S and antibiotic resistance genes are highlighted in Table S1 which highlights the cgMLST type linked to patient metadata, the presence of antibiotic resistance and virulence genes and the MIC values for our isolates. A summary of the antibiotic susceptibility patterns for our isolates is highlighted in Fig S1. MLST identified 21 distinct strains. Amongst these, three strains were classified as ST13, two as ST200, and the remaining 19 were unique singletons. The beta-lactamases *bla*_CTX-M_, *bla*_TEM_ and *bla*_SHV_ genes were present in 71% (*n*=17), 42% (*n*=10) and 100% (*n*=24) isolates, respectively. The *bla*_CTX-M-15_ ESBL was observed in 64% (*n*=16) of isolates, whereas *bla*_CTX-M-14_ was present in just one isolate. Seven isolates had *bla*_CTX-M_ on plasmids ranging from 1.6 to 96 kb, with two also possessing a chromosomal copy (Table S2). The *bla*_SHV-12_, *bla*_SHV-187*_ (+238S, 240K) and *bla*_DHA-1_ ESBLs occurred in five isolates. The *bla*_SHV-12_ was flanked by IS*26* on either side (Table S1B). Additionally, the *bla*_TEM_ was present in ten isolates, *bla*_OXA_ in eight isolates and *bla*_DHA-1_ in two isolates, respectively. The colistin resistance gene *mcr-9.1* was present on 2.5 and 203 kb plasmids in two isolates (Table S2). There were no colistin resistance mutations in the *mgrB* genes observed in our isolates. The *dfrA* gene conferring resistance to trimethoprim was observed in 83% of isolates (*n*=20), whereas the *sul* gene was present in 88% of isolates (*n*=21). Some isolates had as many as three to four copies of the *sul* gene. The macrolide 2′-phosphotransferase I (*mphA*) was observed in five isolates, whereas one isolate had an integron-encoded erythromycin esterase *ereA2*. All isolates except two had at least one aminoglycoside resistance gene, with *strA* (*n*=17) and *strB* (*n*=16) being the most common genes highlighting their role in streptomycin inactivation. The aminoglycoside *N*-acetyltransferases (AAC) belonging to the GCN5-related *N*-acetyltransferase superfamily were the second most abundant class of enzymes with genes encompassing *aac(6')-IIc* (*n*=2) (resistance to GEN but not amikacin), *aac(6')-Ib-cr* (*n*=7), *aac(6')-Ib* (*n*=1) (resistance to TOB, kanamycin and amikacin) and *aac(3)-IIg* (*n*=2), *aac(3)-IIa* (*n*=3) and *aac(3)-IId* (*n*=1) [resistance to GEN, netilmicin, TOB, sisomicin, 2′-N-ethylnetilmicin, 6′-N-ethylnetilmicin and dibekacin]. It is important to note that the *aac(6')-Ib-cr* confers resistance to both aminoglycoside and fluoroquinolone antibiotics through fluoroquinolone-acetylating activity. The aminoglycoside 3′-phosphotransferase *aph(3')-Ia* was observed in four isolates. The *aadA* or *ant(3'')-Ia* gene, which confers resistance to streptomycin and spectinomycin via an adenylyltransferase enzyme, was observed in eight isolates. The *tet*(A) efflux proteins (*n*=7) were the most prevalent, followed by *tet*(D) (*n*=4) and *tet*(B) (*n*=1) genes. Six isolates were positive for chloramphenicol acetyltransferase (*cat*), and 17 were positive for the *qnr* gene, with one isolate also carrying the GyrA-83F, -87A and ParC-80I mutations associated with fluoroquinolone resistance. The yersiniabactin (*ybt*) loci were present in eight isolates, 22 were positive for *wzi* and the K locus and 24 were positive for the O locus with O1 being the most prevalent (Table S1B). None of our isolates had colibactin (*clb*), salmochelin (*iro*), aerobactin (*iuc*) or hypermucoidy (*rmpA* and *rmpA2*) genes.

### Plasmids and antimicrobial gene analyses

**Fig. 1.**
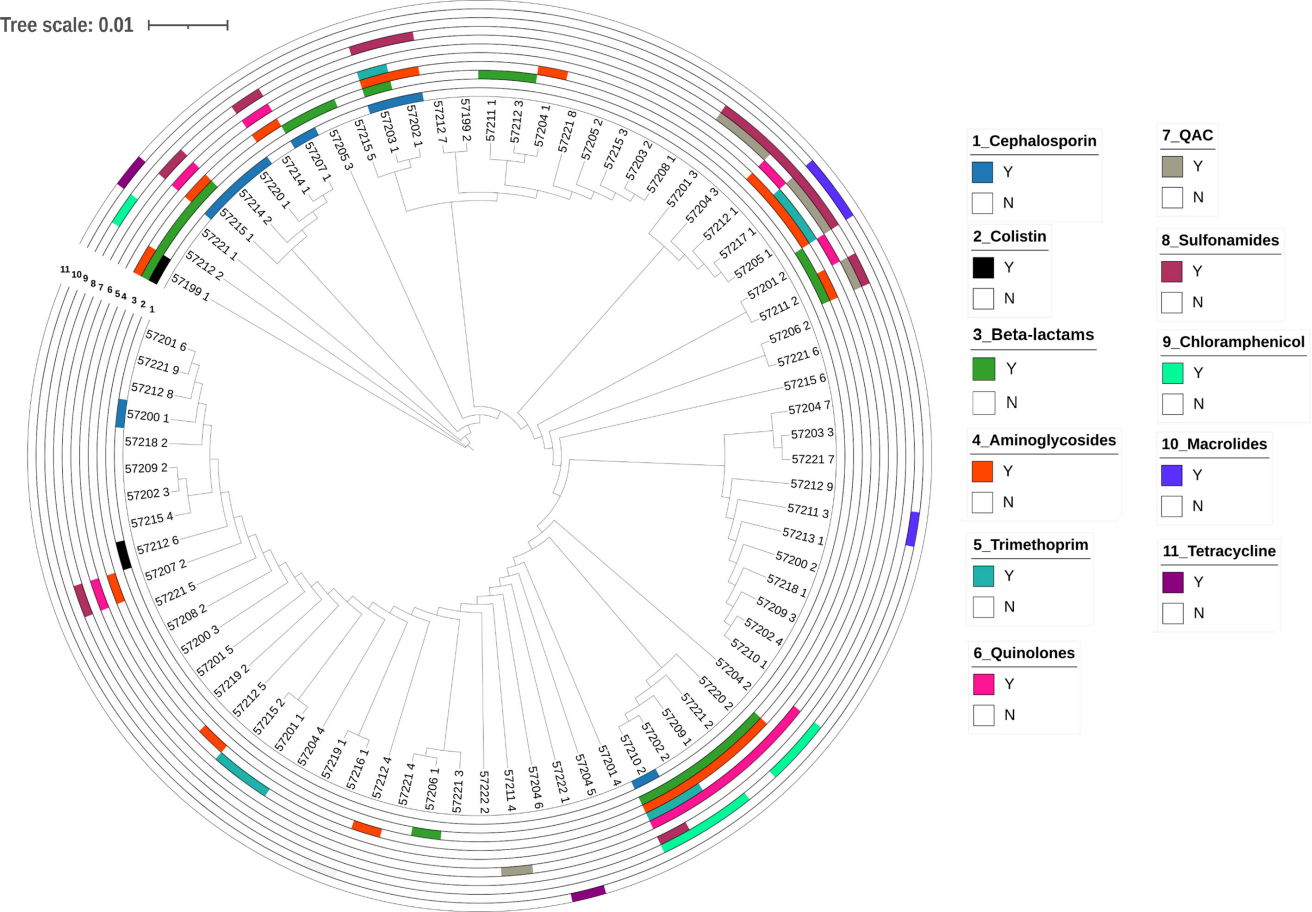
Phylogenetic tree of *Klebsiella* spp. highlighting genetic relatedness and the distribution of antibiotic resistance genes on plasmids.

Table S2 shows the presence of different plasmid-borne AMR determinants present in our isolates. All isolates were positive for at least one plasmid ranging from 1,003 to 203,140 bp. There were 13 isolates having IncFIB(K), which was the most abundant replicon type (Table S2). The phylogenetic relationship clustering between similar plasmids can be observed in Fig. 1 highlighting their role in the dissemination of antimicrobial-resistant genes.

### Genome analysis of isolates from England and Aberdeen, Scotland

**Fig. 2.**
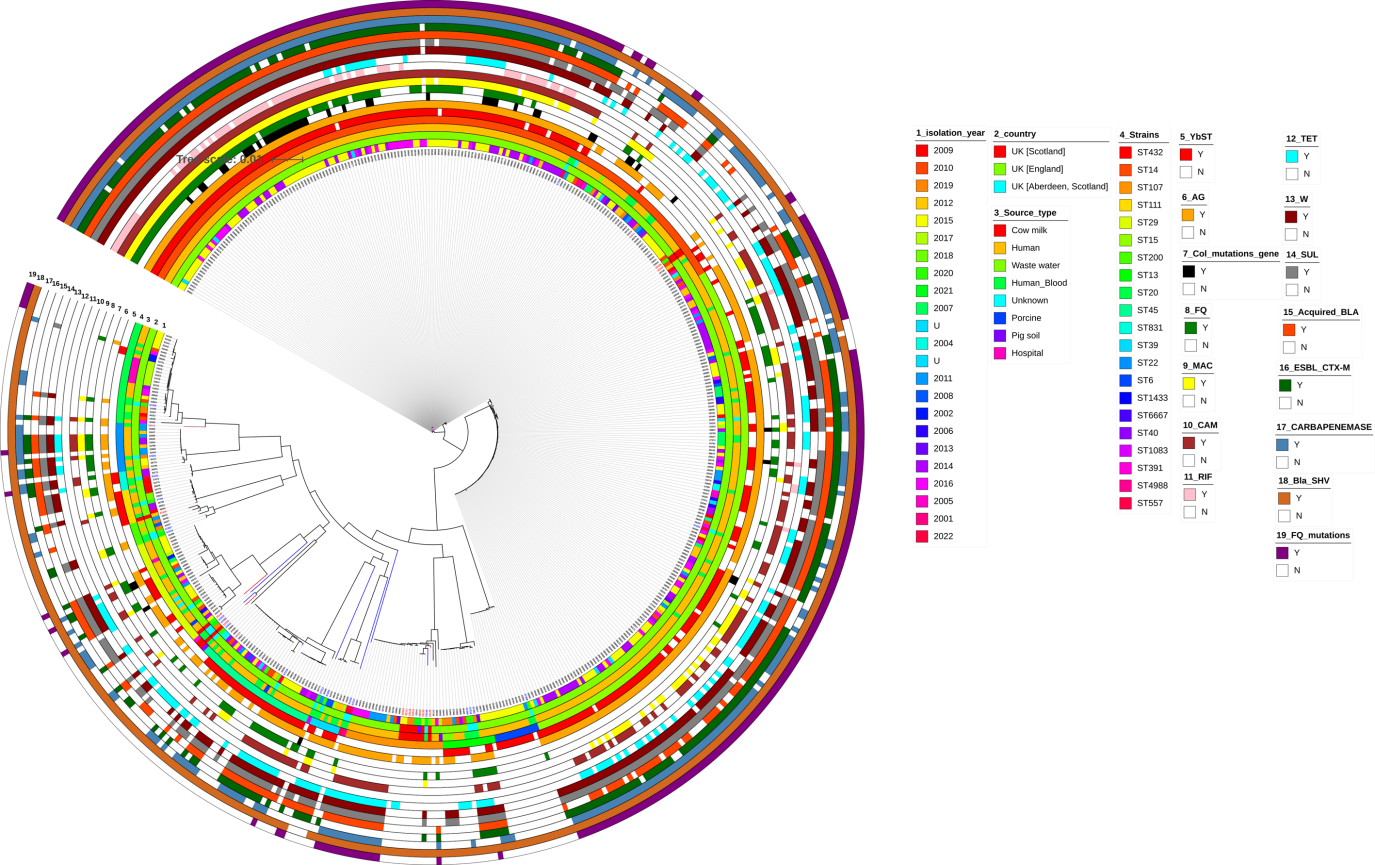
Phylogenetic tree of all *K. pneumoniae* isolates highlighting genetic relatedness and the distribution of antibiotic resistance and virulence determinants (‘Y’ indicates presence, and ‘N’ indicates absence of gene/mutation. YbST, yersiniabactin sequence type; AG, aminoglycoside resistance genes; Col_mutations_gene, colistin mutations or presence of *mcr* gene; FQ, fluoroquinolone resistance-conferring genes; MAC, macrolide resistance genes; CAM, chloramphenicol resistance genes; RIF, rifampicin resistance genes; TET, tetracycline resistance genes; W, trimethoprim resistance genes; SUL, sulphonamide resistance genes; Acquired_BLA, acquired beta-lactamases; ESBL_CTX-M, ESBL gene conferring resistance to third-generation cephalosporins such as cefotaxime; Carbapenemase, gene conferring resistance to carbapenem class of antibiotics; Bla_SHV, intrinsic ESBL found in *Klebsiella* spp.; FQ_mutations, fluoroquinolone resistance-conferring mutations).

**Fig. 3.**
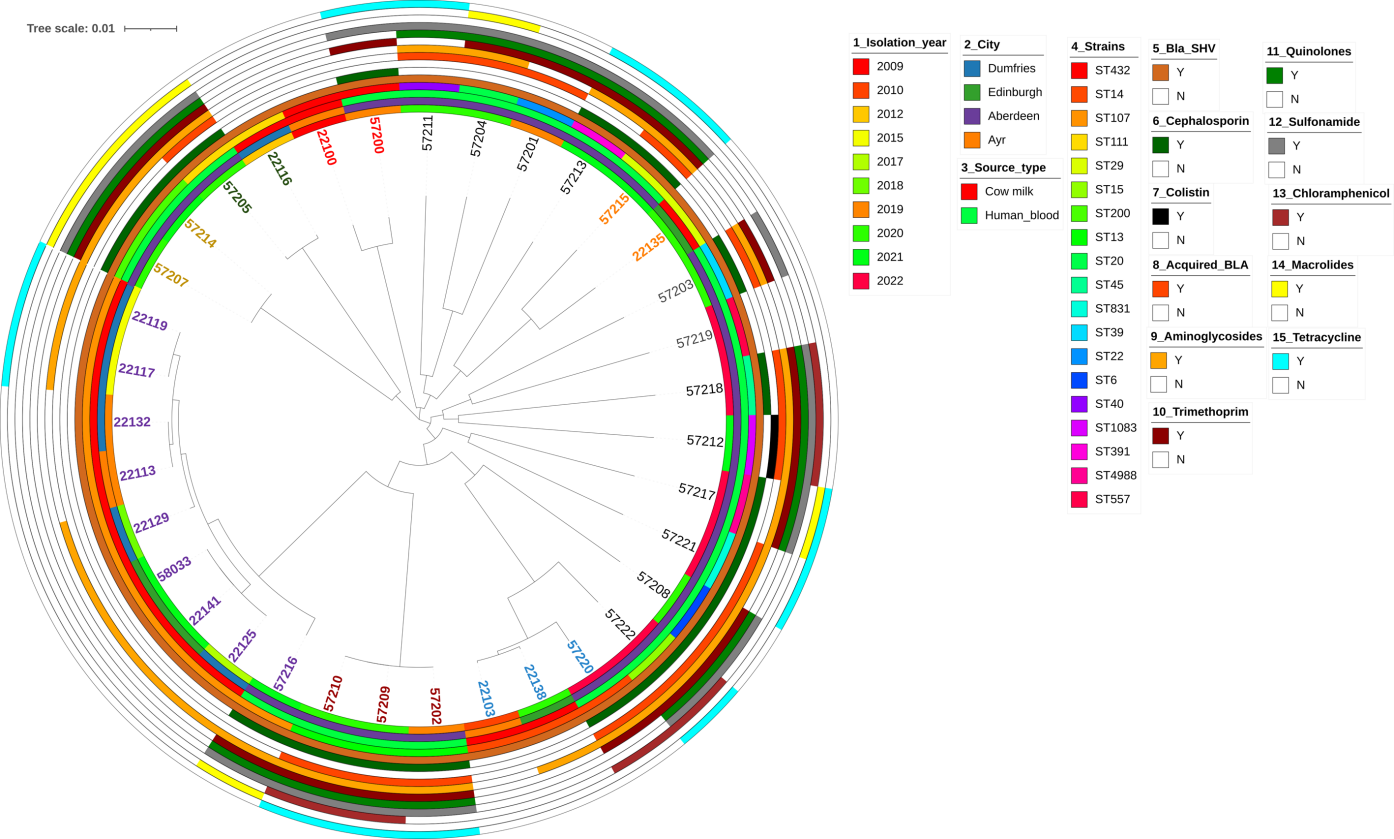
Phylogenetic tree of all *K. pneumoniae* isolates from Scotland highlighting genetic relatedness and the distribution of antibiotic resistance genes (‘Y’ indicates presence, and ‘N’ indicates absence of gene/mutation. Bla_SHV, intrinsic ESBL found in *Klebsiella* spp.; Cephalosporin, presence/absence of resistance to cephalosporin; Colistin, presence/absence of resistance to resistance to colistin; Acquired_BLA, presence/absence of acquired beta-lactamases; AG, presence/absence of resistance to aminoglycosides; W, presence/absence of resistance to trimethoprim; Quinolones, presence/absence of resistance to quinolones; Sulphonamides, presence/absence of resistance to sulphonamides; CAM, presence/absence of resistance to chloramphenicol; MAC, presence/absence of resistance to macrolides; TET, presence/absence of resistance to tetracyclines).

Four hundred and eighty-seven *Klebsiella* isolates of the same sequence type as our 24 isolates, retrieved from BIGSdb (accessed 19 July 2023), provided an overview of resistance types in *K. pneumoniae* across the UK. The phylogenetic relationship between the *K. pneumoniae* isolates can be observed in Fig. 2. It is interesting to note that two isolates, one from cow (ID 2100) and the other from humans (ID 57200), which were ST432, were closely associated, which might link towards an environmental link between zoonotic disease transmission from animals to human infection (Fig. 2). A quick search on *Klebsiella* spp. BIGSdb identified 50 ST432 isolates (last accessed 2 March 2024), some of which were associated with seawater in Ireland, as well as plants and pigs in Italy. Additionally, ST432 has been isolated from a cloacal swab of poultry in Ghana. Whilst isolate 2100 was not resistant, it is possible that resistance due to hospital environmental conditions plays an important role in driving antibiotic resistance in such isolates which could otherwise be sensitive in the natural environments due to lack of antibiotic selective pressure. The overall per cent prevalence of resistance and virulence genes across all 511 isolates is listed in Table 2 which indicates that 60% of our isolates had the *ybt* virulence locus genes, and almost all of them had the *wzi*, K and O loci. It was observed that 77% (*n*=396) isolates had at least one aminoglycoside resistance gene; 35% (*n*=178) of the isolates had a *qnr*-associated fluoroquinolone resistance gene; 59% (*n*=302) of the isolates had at least one mutation in the quinolone resistance determining regions; 70% were resistant to sulphonamides (*n*=360) and 66% to trimethoprim (*n*=337); 65% of the isolates had at least one acquired beta-lactamase (*bla*_OXA_, *bla*_TEM_, *bla*_SCO_, *bla*_DHA_ and *bla*_CMY_) (*n*=332); 99% of isolates had the intrinsic *bla*_SHV_ (*n*=506); and 61% had a carbapenemase (*n*=314) which was exclusively present in isolates across England. It is important to note that we only observed colistin-conferring mutations in *mgrB* (*n*=35), *pmrB* (*n*=11) or the presence of *mcr9.1* (*n*=5) in 10% of the isolates (*n*=49). There were a total of 43 unique clonal groups across the 511 isolates with 32% belonging to CG14 (*n*=164), 29% to CG15 (*n*=146), 6% to CG45 (*n*=31), 4% to CG107 (*n*=22), 4% to CG20 (*n*=21) and 25% to other CGs (*n*=127) (data not shown). It is important to note that strains ST14 (34%, *n*=174) and ST15 (29%, *n*=146) are single-locus variants and were the most predominant strains. Analyses of *bla*_SHV_ showed that SHV-28 variants were the most common (43%, *n*=222) and SHV-12 being an ESBL that was most predominant (2%, *n*=9). The *bla*_NDM-1_ was the most common carbapenemase gene observed in our isolates (24%, *n*=121). The GyrA-83Y, GyrA-87G, ParC-80I and GyrA-83F, GyrA-87A and ParC-80I were the most common mutations observed mostly in strains ST14 (77%, *n*=134/174) and ST15 (96%, *n*=140/146). Forty-one per cent of ST14 and ST15 strains were positive for *bla*_CTX-M-15_. It was also observed that NDM1 was present in 16% of ST14 (*n*=84) and 6% of ST15 (*n*=32), respectively, whilst VIM-4 was also seen in 9% of ST15 (*n*=46). The *bla*_OXA-48_ gene was present in 9% (*n*=46) of isolates and was dispersed across different strains including ST14, ST15, ST29, ST111, ST20, ST45 and ST831. The KL2 locus responsible for the hypervirulence phenotype was present in ST14 (*n*=37, 27%), ST15 (*n*=3) and ST39 (*n*=2) isolates. The virulence factors and antibiotic resistance determinants mapped alongside 13 Scottish isolates with similar sequence types, retrieved from BIGSdb, can be observed in Table S3. It is interesting to note that whilst all our isolates were blood cultures, other similar strains from Scotland were solely isolated from cow milk and did not have the presence of many antibiotic or virulence determinants (Figs3  4).

**Fig. 4.**
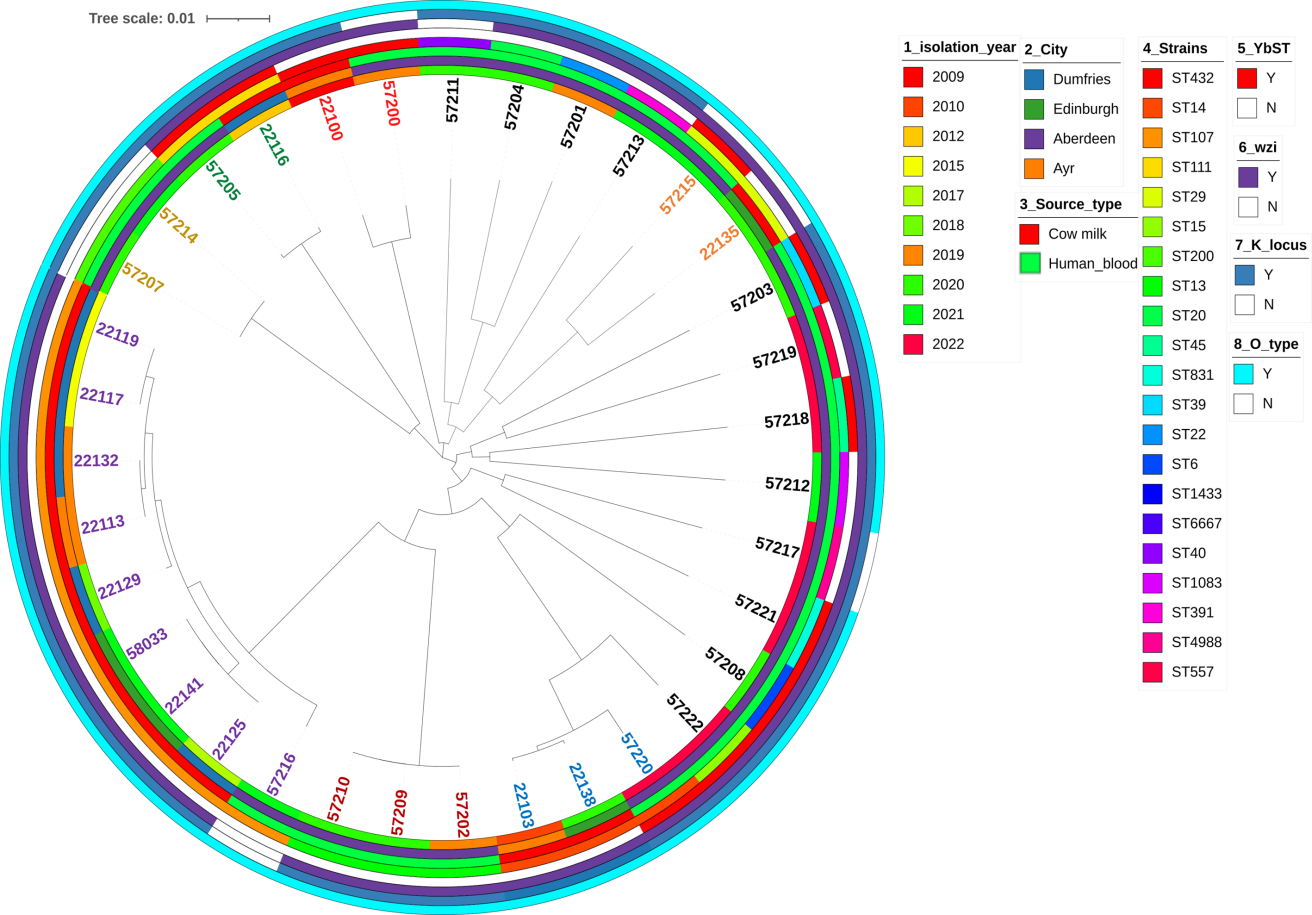
Phylogenetic tree of all *K. pneumoniae* isolates from Scotland highlighting genetic relatedness and the distribution of virulence determinants. (‘Y’ indicates presence, and ‘N’ indicates absence of gene/mutation. YbST, yersiniabactin sequence type; wzi, involved in coding for outer membrane proteins that facilitate the attachment of capsular polysaccharide; K_locus, capsular polysaccharide cluster type; O_type, component of the lipopolysaccharide or O antigen).

**Table 2.** Per cent prevalence of resistance and virulence across isolates from England and Aberdeen, Scotland

Resistance/virulence determinants	N	%
YbST	308	60%
wzi	506	99%
K_locus	511	100%
O_locus	511	100%
O_type	511	100%
Aminoglycosides	395	77%
Colistin_mutations/gene	48	9%
Fluoroquinolone resistance genes	178	35%
Fluoroquinolone_mutations	302	59%
Macrolide	199	39%
Chloramphenicol	280	55%
Rifampicin	73	14%
Sulphonamides	360	70%
Tetracycline	184	36%
Trimethoprim	337	66%
Acquired beta-lactamase	332	65%
Cephalosporin	292	57%
Carbapenems	314	61%
Bla_SHV	506	99%
OMP_mutations	218	43%

## Discussion

In England, cases of *Klebsiella* spp. bacteraemia have risen by 21% between 2017 and 2023. Despite the UK government’s 2017 NHS improvement plan to reduce Gram-negative infections by 50% by 2021, 16% (22 out of 137) of NHS trusts reported increases of over 50%, with at least six trusts seeing cases more than double, and one experiencing a fourfold rise. This underscores the urgent need for enhanced surveillance and robust antimicrobial stewardship (AMS) to prevent *Klebsiella* spp. BSIs, which are the second leading cause of BSI after *E. coli*. Cephalosporin-resistant *K. pneumoniae* has been listed as a critical priority pathogen in 2019 as well as the list that was republished by the WHO in 2024 [8]. The KpSC has developed significant resistance to cephalosporins and other antibiotics, facilitating the spread of resistance genes through horizontal gene transfer. This process drives the emergence and evolution of high-risk clones, which dominate in hospital environments and contribute to outbreaks [27,28]. Furthermore, *Klebsiella* species are ubiquitous and can occur in a range of sources including humans, animals, natural environments and foods [2,29]. The *bla*_CTX-M_ enzymes were initially reported in the second half of the 1980s with the first *bla*_CTX-M_ (CTX-M-1/MEN-1) characterized in *E. coli* strains isolated from German and Italian patients [30,31]. It has been claimed earlier that this variant has probably been derived from the chromosomal beta-lactamase of *K. oxytoca* [32]. It is because it has been observed earlier that the *β*-lactamase *bla*_KLUA_ of *K. ascorbate* is probably the progenitor of only some plasmid-associated CTX-M-type enzymes with IS*Ecp1* responsible for gene mobilization via plasmids and transposition [33]. The CTX-M enzymes are grouped into CTX-M-1, CTX-M-2, CTX-M-8, CTX-M-9 and CTX-M-25 clusters/group, as well as the newly described CTX-M-151 group [34]. The CTX-M-15 (CTX-M group 1) variant was first discovered in 2000 in isolates from urinary and pulmonary human specimens from the Batra Hospital and Medical Research Centre in New Delhi [34,35]. The Asp240Gly substitution in CTX-M-3 is what leads to the formation of CTX-M-15, and this change leads to increased catalytic activity against ceftazidime [36]. The CTX-M-14 and CTX-M-15 genes are widely common in different species of Gram-negative bacteria of the *Enterobacteriaceae* family such as *E. coli*, *K. pneumoniae* and *Proteus mirabilis,* whereas its prevalence in *A. baumannii* and *S*. marcescens remains low across the world [37]. Since the early 2000s, *E. coli* isolates with the CTX-M-15 ESBL are widely distributed across the UK. Heterogeneity of *K. pneumoniae* strains has been reported in several countries such as China, India, Brazil and the USA, which was also observed in our study [28,38,40]. In the Scottish dataset, ST13 was predominantly associated with humans, whereas ST432, ST14, ST107 (predominant strain), ST111 and ST29 were associated with cattle, highlighting the possible route for zoonotic disease transmission. Our study confirms the presence of high-risk clones ST14 and ST15, which are now prevalent in different parts of the world, leading to niche evolution, which can be defined as clones evolving in a specific environment that may have a common ancestor. ST15 was observed in at least 26 European countries and 34 others across the globe, ST14 was prevalent in at least 17 European countries and 34 worldwide and ST45 was present in 21 European countries and 29 across the globe (data not shown). *K. pneumoniae* sequence types ST14 and ST15 are globally recognized as high-risk clones associated with multidrug resistance and significant clinical implications. Previous research highlights the independent evolution of clonal groups 14 (CG14) and 15 (CG15), encompassing ST14 and ST15 and their association with specific capsular types, and the acquisition of antibiotic resistance genes via diverse plasmids [41]. ST15 producing KPC-2, SHV-106 and CTX-M-15 was previously isolated from Anhui, China, which highlights the public health risk posed by the widespread emergence of this high-risk clone [42]. Other than ST14, which has been isolated from neonatal infections in Tanzania, these clones carry multidrug-resistance genes across multiple plasmid replicons, emphasizing their role in invasive infections amongst neonates and their persistence in clinical settings over time [43]. It was observed earlier that *mcr-1* confers low-level colistin resistance, but *mcr-9*-positive strains do not confer colistin resistance. This was also observed in our isolates which were colistin-sensitive despite the presence of *mcr9.1*. The presence of yersiniabactin-associated loci was more common compared to other virulence-associated genes. This shows the ability of bacteria to thrive in the host environment, where iron is typically sequestered by host proteins as a defence mechanism against infection. The O1 serotype was more prevalent in our strains, highlighting its role in virulence [46]. Hypervirulence is thought to be a multifactorial trait resulting from the presence of various virulence genes located on large plasmids, some of which also carry resistance genes. The K locus is involved in the biosynthesis of the capsule polysaccharide with K1 and K2 associated with HvKp linked to increased virulence [, ]. This was predominantly observed in K2-positive ST14 multidrug-resistant isolates in our study, indicating a convergent genotype. Whilst most of the strains had the CTX-M gene present on the chromosome providing a stable mechanism of antibiotic resistance, it was observed that *bla*_CTX-M_ was also present on plasmids, highlighting its major role in the dissemination of antibiotic resistance genes.

## Conclusion

There has been only one previous study on the molecular epidemiology and prevalence of *bla*_CTX-M_ in *K. pneumoniae* in Scotland which focused on the emergence and spread of hospital- and community-acquired CTX-M enzymes [15]. This study, amongst the first in Scotland using a whole-genome sequencing approach for *K. pneumoniae*, offers insights into the molecular dynamics and population structure of CTX-M-15-producing *K. pneumoniae* strains in the UK. It highlights key concerns regarding antibiotic resistance, horizontal gene transfer and the potential role of animal reservoirs in transmission, reinforcing the need for a One Health approach to tackling AMR. Additionally, the study underscores the higher prevalence and evolution of high-risk, MDR-KP clones in England compared to Scotland, likely influenced by differences in population density and demographic shifts. Given England’s higher burden of infectious diseases, these findings emphasize the necessity for tailored, country-specific public health interventions to effectively monitor, manage and control the spread of MDR-KP across the UK. AMS plays a critical role in infection control by reducing the spread of resistant pathogens through responsible antibiotic use and integrating infection prevention measures, such as targeted therapy and early detection. By optimizing antibiotic management, AMS helps preserve the effectiveness of these vital drugs, reduces healthcare costs and supports global public health efforts to combat antimicrobial resistance related to *K. pneumoniae*.

## Supplementary material

10.1099/mgen.0.001413 Uncited Supplementary Material 1.

## Abbreviations

AMR: antimicrobial resistance
AMS: antimicrobial stewardship
BIGSdb: Bacterial Isolate Genome Sequence Database
BSIs: bloodstream infections
ESBL: extended-spectrum *β*-lactamase
GEN: gentamicin
hvKP: hypervirulent *Klebsiella pneumoniae*
ICEs: integrative conjugative elements
iTOL: Interactive Tree of Life
KpSC: *Klebsiella pneumoniae* species complex
MDR-KP: multidrug-resistant *Klebsiella pneumoniae*
PAVs: presence and absence of variants
TOB: tobramycin
WGS: whole-genome sequencing
WHO: World Health Organization
