# Supplementary material for: Molecular epidemiology of extended-spectrum beta-lactamase-producing Klebsiella pneumoniae bloodstream infections from Aberdeen, Scotland, and their comparison to isolates from England
Source: Microb Genom. 2025 Jun 24;11(6):001413. doi: 10.1099/mgen.0.001413 (PMC12186822; doi:10.1099/mgen.0.001413)
Supplement: Uncited Supplementary Material 1. [file mgen-11-01413-s001.pdf]

Supplementary Table S1: Isolate metadata, antimicrobial susceptibilities to different classes of antibiotics and the presence and absence of antibiotic resistance and virulence genes in *Klebsiella* spp isolated from Aberdeen, Scotland.

A: BIGSdb ids with patient meta-data, ST and scgST type (core genome MLST)

B: Antibiotic resistance and virulence genes. BL= beta-lactamase, SHV= SHV genes including SHV-12 and SHV-187* ESBL, Cep= Cepharosporinase, AG=aminoglycoside resistance genes, FQ= Fluoroquinolone resistance conferring mutations/genes, W=Trimethoprim resistance genes, Sul=Sulfonamide resistance genes, Tet=Tetracycline resistance genes, YbST=yersiniabactin sequence type, WZI=outer membrane protein, K= capsular polysaccharide locus, O=lipopolysaccharide. NB: Colistin resistance genes were observed in 57199, 57212, Fluoroquinolone resistance mutation GyrA-83F, GyrA-87A, ParC-80I was observed in 57222.

C: Minimum inhibitory concentration values (expressed as mg/L) of *Klebsiella* isolates from Aberdeen, Scotland. Ampicillin= AP, Co-amoxiclav= AMC, Piperacillin/tazobactam= PTZ, Temocillin= TEM, Cefoxitin= FOX, Cefuroxime= CXM, Cefotaxime= CTX, Ceftazidime= CAZ, Gentamicin= GM, Tobramycin= TN, Amikacin= AK, Ciprofloxacin= CIP, Trimethoprim= TM, Tigecycline= TGC, Colistin= CO. red=resistant, black=sensitive, green=intermediate, NT=not-tested. All ertapenem MICs were <=0.12 mg/L and Meropenem were <=0.25 mg/L

A)

**BIGSdb id**	**Year**	**Organism**	**Age**	**Sex**	**ST**	**scgST (scgMLST629_S)**
57199	2019	*Klebsiella variicola* subsp. *variicola*	71	M	1433	35606
57200	2019	*Klebsiella pneumoniae*	59	M	432	35607
57201	2019	*Klebsiella pneumoniae*	55	M	22	35608
57202	2019	*Klebsiella pneumoniae*	59	M	13	1141
57203	2020	*Klebsiella pneumoniae*	77	M	39	35609
57204	2020	*Klebsiella pneumoniae*	70	M	20	35610
57205	2020	*Klebsiella pneumoniae*	69	M	111	35611

BIGSdb id	Year	Organism	Age	Sex	ST	scgST (scgMLST629_S)
57206	2020	*Klebsiella quasipneumoniae* subsp. *similipneumoniae*	66	F	6667	35612
57207	2020	*Klebsiella pneumoniae*	61	M	200	35613
57208	2020	*Klebsiella pneumoniae*	63	M	6	35614
57209	2020	*Klebsiella pneumoniae*	77	F	13	35615
57210	2020	*Klebsiella pneumoniae*	82	F	13	35616
57211	2020	*Klebsiella pneumoniae*	50	M	40	35617
57212	2021	*Klebsiella pneumoniae*	57	M	1083	35618
57213	2021	*Klebsiella pneumoniae*	83	M	391	35619
57214	2021	*Klebsiella pneumoniae*	40	F	200	35620
57215	2021	*Klebsiella pneumoniae*	70	M	29	35621
57216	2021	*Klebsiella pneumoniae*	62	F	107	35622
57217	2022	*Klebsiella pneumoniae*	77	M	4988	35623
57218	2022	*Klebsiella pneumoniae*	54	F	45	35624
57219	2022	*Klebsiella pneumoniae*	59	F	557	35625
57220	2022	*Klebsiella pneumoniae*	82	M	14	35626
57221	2022	*Klebsiella pneumoniae*	72	F	831	35627
57222	2022	*Klebsiella pneumoniae*	70	M	15	35628

B)

BIGSdb id	BL	Intrinsic SHV	Cep	AG	FQ	W	Sul	Tet	YbST	wzi	K_locus	O_type
57199	TEM-1D, LEN-2	SHV-12	IS26 SHV-12 IS26	aac(3)-IIg, aac(6')-IIc, strA [APH(3'')-Ib] strB [aph(6)-Id]		dfrA19 (homolog)	sul1	tet(D)	224	wzi32	KL31	O3/O3a
57200		SHV-60	CTX-M-14			dfrA1	sul1	tet(A)		wzi149	KL62	O2a
57201		SHV-1	DHA-1		qnrB4	dfrA1	sul1, sul1			wzi238	KL11	O3b
57202	OXA-1	SHV-1	CTX-M-15	aac(3)-IIa, aac(6')-Ib-cr, strA, strB	qnrB1	dfrA14 (homolog)	sul2	tet(A)		wzi40	KL3	O1
57203	TEM-1D	SHV-11	CTX-M-15	strA, strB		dfrA14 (homolog)	sul2		294-1LV	wzi2	KL2 (KL30)	O1
57204	TEM-1D, OXA-9	SHV-187*, SHV-12	SHV-187*, SHV-12	aac(6')-Ib' (homolog), aadA (homolog)	qnrA1	dfrA5	sul1, sul1			wzi84	KL28	O1
57205	TEM-1D	SHV-11	CTX-M-15	aadA2, aph3-Ia, strA (homolog), strA (homolog)	qnrS1	dfrA12	sul1, sul2, sul2		147	wzi63	KL63	O1
57206	OKP-B-7	SHV-1	None	aadA			sul1	tet(D)				O3/O3a
57207		SHV-1	CTX-M-15, CTX-M-15	aadA2, strA (homolog), strB (homolog)	qnrS1	dfrA12	sul1, sul2			wzi39	KL39 (KL13)	O3b
57208	TEM-1D,	SHV-1	CTX-M-15	strA, strB	qnrB1	dfrA14 (homolog)	sul2		376	wzi7	KL7	O1
57209	OXA-1	SHV-1	CTX-M-15	aac(6')-Ib-cr, strA, strB	qnrB1	dfrA14 (homolog)	sul2	tet(A)		wzi40	KL3	O1

BIGSdb id	BL	Intrinsic SHV	Cep	AG	FQ	W	Sul	Tet	YbST	wzi	K_locus	O_type
57210	OXA-1	SHV-1	CTX-M-15	aac(6')-Ib-cr, strA, strB	qnrB1	dfrA14 (homolog)	sul2	tet(A)		wzi40	KL3	O1
57211		SHV-11, SHV-11	DHA-1	strA, strA, strB, strB	qnrB4		sul1, sul2 (homolog), sul2 (homolog)	tet(D)		wzi173	KL102 (KL149,KL 155)	unknown (OL101)
57212	TEM-1D	SHV-11, SHV-12	SHV-12	aac(3)-IIg, aac(6')-IIc, aadA2, aph(3')-Ia (homolog, fragment), strA, strB	qnrB2	dfrA19 (homolog)	sul1, sul1, sul1, sul2			wzi462	KL?	O1
57213		SHV-11	CTX-M-15	aac(3)-IId, strA (homolog), strB (homolog)	qnrS1	dfrA14 (homolog)	sul2	tet(D)		wzi554	KL?	O1
57214		SHV-1	CTX-M-15, CTX-M-15	aadA2, strA (homolog), strB (homolog)	qnrS1	dfrA12	sul1, sul2			wzi39	KL39 (KL13)	O3b
57215	TEM-1D	SHV-187	CTX-M-15	strA, strB	qnrS1	dfrA14 (homolog)	sul2	tet(A)	417-2LV	wzi115	KL54	O1
57216		SHV-1	CTX-M-15	aadA2, aph3-Ia, strA (homolog), strB (homolog)	qnrS1	dfrA12	sul1, sul2					O1
57217		SHV-28 (homolog)	CTX-M-15	aadA2, aph3-Ia, strA (homolog), strB (homolog)	qnrS1	dfrA12	sul1, sul2	tet(A)		wzi7	KL7	O2afg

BIGSdb id	BL	Intrinsic SHV	Cep	AG	FQ	W	Sul	Tet	YbST	wzi	K_locus	O_type
57218	TEM-1D,OXA-1	SHV-1	CTX-M-15	aac(3)-IIa, aac(6')-Ib-cr, strA, strB	qnrB1	dfrA14 (homolog)	sul2			wzi202	KL127 (KL155)	unknown (OL101)
57219		SHV-1	None							wzi135	KL3	O1
57220	OXA-1	SHV-28	CTX-M-15	aac(6')-Ib-cr		dfrA14 (homolog)			183-2LV	wzi2	KL2 (KL30)	O1
57221	TEM-1D,OXA-1	SHV-11	CTX-M-15, CTX-M-15	aac(6')-Ib-cr				tet(B) (homolog), tetR	89-2LV	wzi18	KL18	O1
57222	TEM-1D,OXA-1 (homolog)	SHV-28	CTX-M-15	aac(3)-IIa, aac(6')-Ib-cr, strA, strB	qnrB1	dfrA14 (homolog)	sul2	tet(A)	17	wzi93	KL112	O1

C)

BIGSdb id	AP	AMC	PTZ	TEM	FOX	CXM	CTX	CAZ	GM	TN	AK	CIP	TM	TGC	CO
57199	>=32.0	16	<=4.0	<=4.0	<=4.0	16	16	32	>=16.0	8	<=2.0	<=0.25	>=16.0	1	0.5
57200	>=32.0	>=32.0	32	<=4.0	<=4.0	>=64.0	>=64.0	8	<=1.0	<=1.0	<=2.0	<=0.25	>=16.0	2	NT
57201	>=32.0	>=32.0	<=4.0	<=4.0	>=64.0	>=64.0	16	>=64.0	<=1.0	<=1.0	<=2.0	<=0.25	>=16.0	<=0.5	NT
57202	>=32.0	>=32.0	32	8	<=4.0	>=64.0	>=64.0	>=64.0	>=16.0	>=16.0	<=2.0	>=4.0	>=16.0	1	NT
57203	>=32.0	16	<=4.0	<=4.0	<=4.0	>=64.0	>=64.0	32	<=1.0	<=1.0	<=2.0	<=0.25	>=16.0	1	NT
57204	>=32.0	>=32.0	32	<=4.0	<=4.0	32	32	>=64.0	8	>=16.0	4	1	>=16.0	1	NT
57205	>=32.0	>=32.0	<=4.0	<=4.0	<=4.0	>=64.0	>=64.0	8	<=1.0	<=1.0	<=2.0	0.5	>=16.0	<=0.5	NT
57206	>=32.0	>=32.0	>=128.0	<=4.0	<=4.0	4	<=0.25	16	<=1.0	<=1.0	<=2.0	<=0.25	<=0.5	1	NT
57207	>=32.0	16	<=4.0	<=4.0	<=4.0	>=64.0	>=64.0	32	<=1.0	<=1.0	<=2.0	1	>=16.0	1	NT
57208	>=32.0	16	8	<=4.0	<=4.0	>=64.0	>=64.0	32	<=1.0	<=1.0	<=2.0	0.5	>=16.0	1	NT
57209	>=32.0	>=32.0	32	<=4.0	<=4.0	>=64.0	>=64.0	>=64.0	>=16.0	>=16.0	<=2.0	>=4.0	>=16.0	2	NT
57210	>=32.0	>=32.0	16	<=4.0	<=4.0	>=64.0	>=64.0	32	<=1.0	8	<=2.0	2	>=16.0	2	NT
57211	>=32.0	>=32.0	>=128.0	<=4.0	>=64.0	>=64.0	16	>=64.0	<=1.0	<=1.0	<=2.0	0.5	<=0.5	1	NT
57212	>=32.0	8	<=4.0	<=4.0	<=4.0	4	16	32	>=16.0	8	<=2.0	<=0.25	>=16.0	<=0.5	0.5
57213	>=32.0	4	<=4.0	<=4.0	<=4.0	>=64.0	>=64.0	8	>=16.0	8	<=2.0	<=0.25	>=16.0	<=0.5	NT
57214	>=32.0	8	<=4.0	<=4.0	<=4.0	>=64.0	>=64.0	32	<=1.0	<=1.0	<=2.0	1	>=16.0	1	NT
57215	>=32.0	16	<=4.0	<=4.0	<=4.0	>=64.0	>=64.0	32	<=1.0	<=1.0	<=2.0	1	>=16.0	2	NT
57216	>=32.0	4	<=4.0	<=4.0	<=4.0	>=64.0	>=64.0	8	<=1.0	<=1.0	<=2.0	1	>=16.0	<=0.5	NT
57217	>=32.0	8	<=4.0	<=4.0	<=4.0	>=64.0	>=64.0	32	<=1.0	<=1.0	<=2.0	1	>=16.0	2	NT
57218	>=32.0	>=32.0	32	<=4.0	<=4.0	>=64.0	>=64.0	32	>=16.0	>=16.0	<=2.0	2	>=16.0	<=0.5	NT
57219	>=32.0	16	8	<=4.0	<=4.0	4	16	4	<=1.0	<=1.0	<=2.0	<=0.25	8	2	NT
57220	>=32.0	>=32.0	>=128.0	<=4.0	<=4.0	>=64.0	>=64.0	>=64.0	<=1.0	>=16.0	8	0.5	>=16.0	1	NT
57221	>=32.0	>=32.0	>=128.0	<=4.0	<=4.0	>=64.0	>=64.0	>=64.0	<=1.0	>=16.0	16	<=0.25	<=0.5	1	NT
57222	>=32.0	>=32.0	32	<=4.0	16	>=64.0	>=64.0	32	>=16.0	>=16.0	<=2.0	>=4.0	>=16.0	>=8.0	NT

Supplementary Table S2: The occurrence of different plasmid types in isolates of *Klebsiella* spp

Resistance conferring genes: Cep=Cephalosporinase, Col= Colistin, AcqBL=Acquired Beta-lactamases, AG=Aminoglycosides, W= Trimethoprim, FQ= Fluoroquinolones, S=Sulfonamides, CAM= Chloramphenicol, MAC=Macrolide, TET=Tetracycline, Isolate_plasmid indicates BIGSdb id followed by plasmid number

Isolate_plasmid	Size (bp)	Plasmid type	Cep	Col	AcqBL	AG	W	FQ	QAC	S	CAM	MAC	TET
57199_1	203140			*mcr-9.1*	*bla*$_{TEM-1B}$	*aph(3'')-Ib, aph(6)-Id*							
57199_2	48850	IncHI2, IncHI2A											
57200_1	1619		*bla*$_{CTX-M-14}$										
57200_2	17741	IncFIB(K)											
57200_3	1699												
57201_1	3639						*dfrA1*						
57201_2	7720				*bla*$_{DHA-1}$			*qnrB4*					
57201_3	1750								*qacE*	*sul1*			
57201_4	7405												
57201_5	1226												
57201_6	8916	Col440I											
57202_1	56291		*bla*$_{CTX-M-15}$			*aph(6)-Id, aph(3'')-Ib*				*sul2*			
57202_2	22041				*bla*$_{OXA-1}$	*aac(3)-IIa, aac(6')-Ib-cr*	*dfrA14*	*qnrB1, aac(6')-Ib-cr*			*catB3, catB3*		
57202_3	43908	IncFII(K)											
57202_4	35824	IncFIB(K)											
57203_1	96405		*bla*$_{CTX-M-15}$		*bla*$_{TEM-1B}$	*aph(3'')-Ib, aph(6)-Id*	*dfrA14*			*sul2*			
57203_2	127592												
57203_3	7721	IncFIB(K)											

Isolate_plasmid	Size (bp)	Plasmid type	Cep	Col	AcqBL	AG	W	FQ	QAC	S	CAM	MAC	TET
57204_1	20121	IncFII(K)			$bla_{\text{TEM-1B}}$, $bla_{\text{TEM-141}}$, $bla_{\text{OXA-9}}$	*aac(6')-Ib*							
57204_2	4548							*qnrA1*			*cmlA1*		
57204_3	1276								*qacE*	*sul1*			
57204_4	2627												
57204_5	3592	Col440I											
57204_6	13928	IncN											
57204_7	12398	IncFIB(K)											
57205_1	13242					*aadA2*	*dfrA12*		*qacE*	*sul1*		*mph*(A)	
57205_2	113708												
57205_3	2156				$bla_{\text{TEM-1C}}$								
57206_1	81899	IncFIB, IncFII			bla_{SHV}								
57206_2	10694	Col156											
57207_1	39059		$bla_{\text{CTX-M-15}}$										
57207_2	9881					*aph(6)-Id*, *aph(3'')-Ib*		*qnrS1*		*sul2*			
57208_1	117830												
57208_2	3642	Col440I											
57209_1	20201				$bla_{\text{OXA-1}}$	*aac(6')-Ib-cr*		*aac(6')-Ib-cr*			*catB3*		
57209_2	57474	IncFII(K)											
57209_3	64675	IncFIB(K)											
57210_1	93733	IncFIB(K), IncFII(K)											
57210_2	34528		$bla_{\text{CTX-M-15}}$		$bla_{\text{OXA-1}}$	*aph(6)-Id*, *aph(3'')-Ib*, *aac(6')-Ib-cr*	*dfrA14*	*qnrB1*, *aac(6')-Ib-cr*		*sul2*	*catB3*		
57211_1	12104				bla_{SHV}								

Isolate_plasmid	Size (bp)	Plasmid type	Cep	Col	AcqBL	AG	W	FQ	QAC	S	CAM	MAC	TET
57211_2	19214				*bla*$_{\text{DHA-1}}$	*aph(3'')-Ib*, *aph(6)-Id*			*qacE*	*sul1*			
57211_3	13364	IncFIB(K)										*ere*(A)	
57211_4	11431	IncFIA(HI1)							*qacE*				
57212_1	69426					*aac(6')-IIc*		*qnrB2*		*sul1*, *sul1*			
57212_2	129439				*bla*$_{\text{TEM-1B}}$						*catA2*		
57212_3	2920				*bla*$_{\text{SHV}}$								
57212_4	24786					*aph(3')-Ia*							
57212_5	1003					*aadA2b*							
57212_6	2558			*mcr-9.1*									
57212_7	35264	IncHI2											
57212_8	3864	IncFIA(HI1)											
57212_9	8260	IncFIB(K)											
57213_1	17048	IncFIB(K)											
57214_1	60451					*aph(3'')-Ib*, *aph(6)-Id*		*qnrS1*		*sul2*			
57214_2	6457		*bla*$_{\text{CTX-M-15}}$										
57215_1	25361		*bla*$_{\text{CTX-M-15}}$		*bla*$_{\text{TEM-1B}}$	*aph(6)-Id*, *aph(3'')-Ib*		*qnrS1*		*sul2*			
57215_2	1971						*dfrA14*						
57215_3	75206												
57215_4	64951												
57215_5	31308	IncFIB(K)											
57215_6	6816	IncY											
57216_1	10716												
57217_1	11601					*aadA2*	*dfrA12*		*qacE*	*sul1*		*mph*(A)	
57218_1	17544	IncFIB(K)											
57218_2	5875												

Isolate_plasmid	Size (bp)	Plasmid type	Cep	Col	AcqBL	AG	W	FQ	QAC	S	CAM	MAC	TET
57219_1	65340												
57219_2	7187	IncFIB(K)											
57220_1	6039		*bla*$_{CTX-M-15}$										
57220_2	2186				*bla*$_{OXA-1}$	*aac(6')-Ib-cr*		*aac(6')-Ib-cr*			*catB3*		
57221_1	11784				*bla*$_{TEM-1B}$								*tet*(B)
57221_2	17867				*bla*$_{OXA-1}$	*aac(6')-Ib-cr*		*aac(6')-Ib-cr*					
57221_3	31457												
57221_4	21985	IncFII											
57221_5	12310	IncFIB											
57221_6	14646	Col156											
57221_7	5007	IncFIB(K)											
57221_8	3941	Col440I											
57221_9	10702	IncFII											
57222_1	5162												*tet*(A)
57222_2	3103												

Supplementary Table S3: Antibiotic resistance and virulence genes in isolates from Aberdeen and the rest of Scotland. Resistance conferring genes: Cep=Cephalosporinase, Col= Colistin, AcqBL=Acquired Beta-lactamases, AG=Aminoglycosides, W= Trimethoprim, FQ= Fluoroquinolones, S=Sulfonamides, CAM= Chloramphenicol, MAC=Macrolide, TET=Tetracycline

id	isolation_year	city	source_type	Chr_ST	YbST	wzi	K_locus	O_type	Intrinsic Beta-lactam	Cep	Col	Acq BL	AG	W	FQ	S	CAM	MAC	TET
22100	2009	Ayr	Cow milk	ST432		wzi130	KL58	O3b	SHV-60										
22103	2010	Ayr	Cow milk	ST14		wzi16	KL16 (KL143)	O1	SHV-28.v1^										
22113	2019	Ayr	Cow milk	ST107		wzi516	KL?	O1	SHV-1^										
22116	2012	Dumfries	Cow milk	ST111	63-2LV	wzi63	KL63	O1	SHV-11.v1^										
22117	2015	Dumfries	Cow milk	ST107		wzi69	KL143 (KL101)	O1	SHV-1^				strA.v1; strB.v1						tet(B).v2 *;tetR

id	isolation_year	city	source_type	Chr_ST	YbST	wzi	K_locus	O_type	Intrinsic Beta-lactam	Cep	Col	Acq BL	AG	W	FQ	S	CAM	MAC	TET
22119	2015	Dumfries	Cow milk	ST107		wzi69	KL143 (KL101)	O1	SHV-1^				strA.v1; strB.v1						tet(B).v2*;tetR
22125	2017	Dumfries	Cow milk	ST107		wzi194	KL108	O1	SHV-1^				strA.v1; strB.v1						
22129	2018	Dumfries	Cow milk	ST107		wzi350	KL113	O1	SHV-1^				strA.v1; strB.v1						
22132	2019	Dumfries	Cow milk	ST107		wzi516	KL?	O1	SHV-1^										
22135	2020	Edinburgh	Cow milk	ST29		wzi85	KL30	O1	SHV-11.v1^										
22138	2020	Edinburgh	Cow milk	ST14		wzi16	KL16 (KL143)	O1	SHV-28.v1^				strA.v1; strB.v1						
22141	2021	Edinburgh	Cow milk	ST107		wzi194	KL108	O1	SHV-1^				strA.v1; strB.v1						
57199	2019	Aberdeen	Human_Blood	ST1433	224	wzi32	KL31	O3/O3a	SHV-12, LEN-2^		mcr-9.1	TEM-1D.v1^	aac(3)-IIg;aac(6')-IIc;strA.v1;strB.v1	dfrA19*		sul1			tet(D)
57200	2019	Aberdeen	Human_Blood	ST432		wzi149	KL62	O2a	SHV-60	CTX-M-14				dfrA1.v1		sul1			tet(A).v1
57201	2019	Aberdeen	Human_Blood	ST22		wzi238	KL11	O3b	SHV-1^			DHA-1		dfrA1.v1	qnrB4	2 (sul1)			
57202	2019	Aberdeen	Human_Blood	ST13		wzi40	KL3	O1	SHV-1^	CTX-M-15		OXA-1	aac(3)-IIa.v1^;aac(6')-Ib-cr.v2;strA.v1^;strB.v1	dfrA14.v2*	qnrB1.v2^	sul2			tet(A).v1
57203	2020	Aberdeen	Human_Blood	ST39	294-1LV	wzi2	KL2 (KL30)	O1	SHV-11.v1^	CTX-M-15		TEM-1D.v1^	strA.v1^;strB.v1	dfrA14.v2*		sul2			
57204	2020	Aberdeen	Human_Blood	ST20		wzi84	KL28	O1	SHV-12, SHV-187*			OXA-9.v1;TEM-1D.v1^	aac(6')-Ib'.v1*;aadA*	dfrA5	qnrA1	sul1;sul1		ereA2	
57205	2020	Aberdeen	Human_Blood	ST111	147	wzi63	KL63	O1	SHV-11.v1^	CTX-M-15		TEM-1D.v1^	aadA2^;aph3-Ia.v1^;strA.v1*;strA.v1*	dfrA12	qnrS1	sul1; 2 (sul2)		mphA	
57206	2020	Aberdeen	Human_Blood	ST6667				O3/O3a	OKP-B-7^;SHV-1^				aadA^			sul1			tet(D)
57207	2020	Aberdeen	Human_Blood	ST200		wzi39	KL39 (KL13)	O3b	SHV-1^	2 (CTX-M-15)			aadA2^;strA.v1*;strB.v1*	dfrA12	qnrS1	sul1;sul2		mphA	

id	isolation_year	city	source_type	Chr_ST	YbST	wzi	K_locus	O_type	Intrinsic Beta-lactam	Cep	Col	Acq BL	AG	W	FQ	S	CAM	MAC	TET
57208	2020	Aberdeen	Human_Blood	ST6	376	wzi7	KL7	O1	SHV-1	CTX-M-15		TEM-1D.v1^	strA.v1^;strB.v1	dfrA14.v2*	qnrB1.v2^	sul2			
57209	2020	Aberdeen	Human_Blood	ST13		wzi40	KL3	O1	SHV-1^	CTX-M-15		OXA-1	aac(3)-IIa.v1^;aac(6')-Ib-cr.v2;strA.v1^;strB.v1	dfrA14.v2*	qnrB1.v2^	sul2	CatB4.v1		tet(A).v1
57210	2020	Aberdeen	Human_Blood	ST13		wzi40	KL3	O1	SHV-1^	CTX-M-15		OXA-1	aac(6')-Ib-cr.v2;strA.v1^;strB.v1	dfrA14.v2*	qnrB1.v2^	sul2	CatB4.v1		tet(A).v1
57211	2020	Aberdeen	Human_Blood	ST40		wzi173	KL102 (KL149,KL155)	unknown (OL101)	SHV-11.v1^;SHV-11.v1^			DHA-1	strA.v1; strA.v1^;strB.v1; strB.v1		qnrB4	sul1; 2 (sul2*)			tet(D)
57212	2021	Aberdeen	Human_Blood	ST1083		wzi462	KL?	O1	SHV-12, SHV-11.v1^		mcr-9.1	TEM-1D.v1^	aac(3)-IIg;aac(6')-IIc;aadA2;aph(3')-Ia*?;strA.v1;strB.v1	dfrA19*	qnrB2.v1^	3 (sul1);sul2	2 (catII.2*)		
57213	2021	Aberdeen	Human_Blood	ST391		wzi554	KL?	O1	SHV-11.v1^	CTX-M-15			aac(3)-IId^;strA.v1*;strB.v1*	dfrA14.v2*	qnrB1.v1	sul2			tet(D)^
57214	2021	Aberdeen	Human_Blood	ST200		wzi39	KL39 (KL13)	O3b	SHV-1^	2 (CTX-M-15)			aadA2^; strA.v1*; strB.v1*	dfrA12	qnrS1	sul1;sul2		mphA	
57215	2021	Aberdeen	Human_Blood	ST29	417-2LV	wzi115	KL54	O1	SHV-187	CTX-M-15		TEM-1D.v1^	strA.v1^;strB.v1	dfrA14.v2*	qnrS1	sul2			tet(A).v1
57216	2021	Aberdeen	Human_Blood	ST107				O1	SHV-1^	CTX-M-15			aadA2^; aph3-Ia.v1^;strA.v1*;strB.v1*	dfrA12	qnrS1	sul1;sul2		mphA	
57217	2022	Aberdeen	Human_Blood	ST4988		wzi7	KL7	O2afg	SHV-28.v1*	CTX-M-15			aadA2^; aph3-Ia.v1^;strA.v1*;strB.v1*	dfrA12	qnrS1	sul1;sul2		mphA	tet(A).v1
57218	2022	Aberdeen	Human_Blood	ST45		wzi202	KL127 (KL155)	unknown (OL101)	SHV-1	CTX-M-15		OXA-1;TEM-1D.v1^	aac(3)-IIa.v1^;a	dfrA14.v2*	qnrB1.v2^	sul2	CatB4.v1		

id	isolation_year	city	source_type	Chr_ST	YbST	wzi	K_locus	O_type	Intrinsic Beta-lactam	Cep	Col	Acq BL	AG	W	FQ	S	CAM	MAC	TET
													ac(6')-Ib-cr.v2;strA.v1^;strB.v1						
57219	2022	Aberdeen	Human_Blood	ST557		wzi135	KL3	O1	SHV-1^										
57220	2022	Aberdeen	Human_Blood	ST14	183-2LV	wzi2	KL2 (KL30)	O1	SHV-28.v1^	CTX-M-15		OXA-1	aac(6')-Ib-cr.v2	dfrA14.v2*			CatB4.v1		
57221	2022	Aberdeen	Human_Blood	ST831	89-2LV	wzi18	KL18	O1	SHV-11.v1^	2 (CTX-M-15)		OXA-1;TEM-1D.v1^	aac(6')-Ib-cr.v2						tet(B).v2*;tetR
57222	2022	Aberdeen	Human_Blood	ST15	17	wzi93	KL112	O1	SHV-28.v1^	CTX-M-15		OXA-1*;TEM-1D.v1^	aac(3)-IIa.v1^;aac(6')-Ib-cr.v2;strA.v1^;strB.v1	dfrA14.v2*	qnrB1.v2^;GyrA-83F;87A;ParC-80I	sul2	CatB4.v1		tet(A).v1
58033	2021	Edinburgh	Cow milk	ST107		wzi194	KL108	O1	SHV-1^				strA.v1;strB.v1						

Supplementary Figure S1: Diagrammatic representation of antibiotic susceptibility patterns of *Klebsiella* spp isolates from Aberdeen, Scotland.

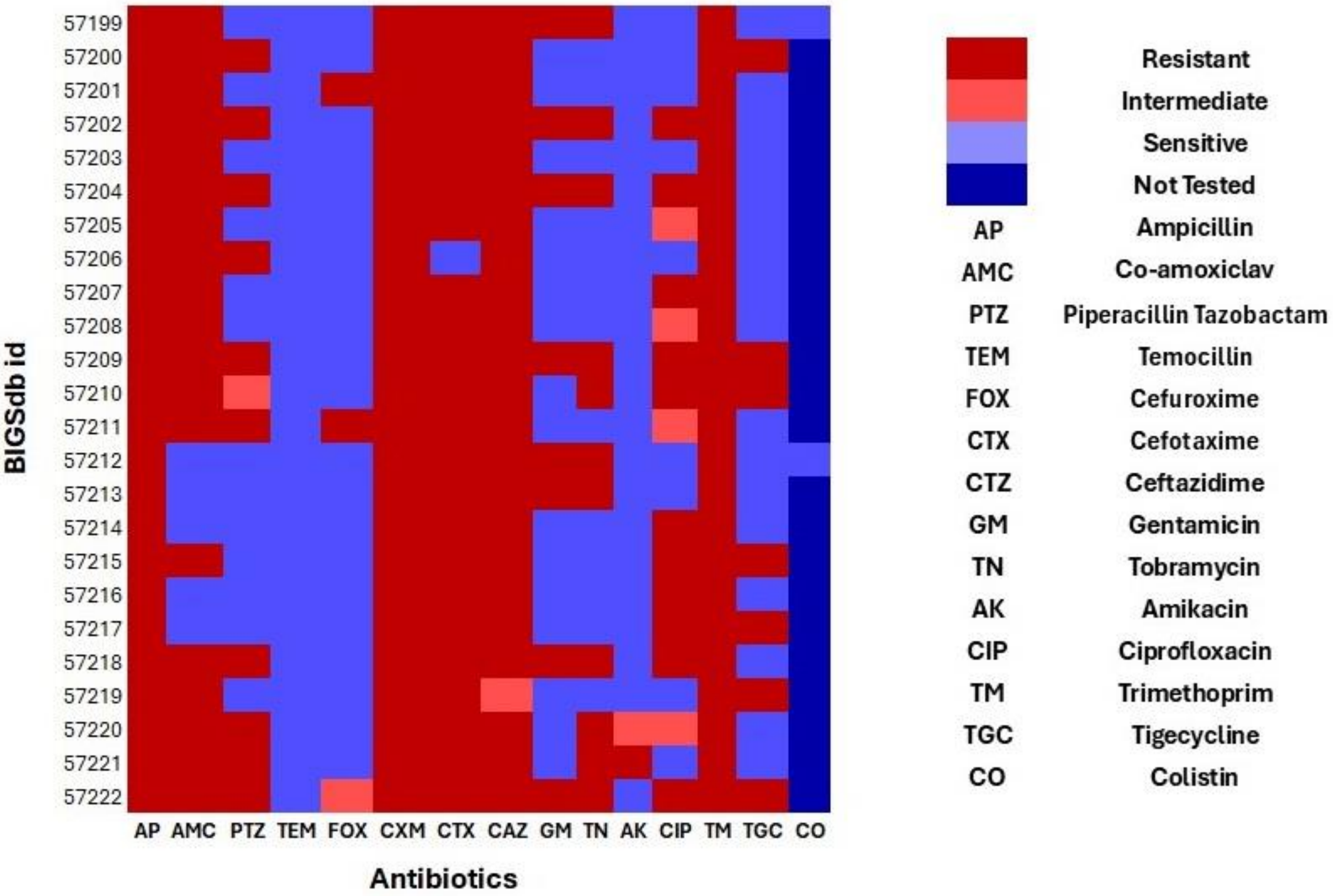
BIGSdb id
57199
57200
57201
57202
57203
57204
57205
57206
57207
57208
57209
57210
57211
57212
57213
57214
57215
57216
57217
57218
57219
57220
57221
57222
AP AMC PTZ TEM FOX CXM CTX CAZ GM TN AK CIP TM TGC CO
Antibiotics
Resistant
Intermediate
Sensitive
Not Tested
AP Ampicillin
AMC Co-amoxiclav
PTZ Piperacillin Tazobactam
TEM Temocillin
FOX Cefuroxime
CTX Cefotaxime
CTZ Ceftazidime
GM Gentamicin
TN Tobramycin
AK Amikacin
CIP Ciprofloxacin
TM Trimethoprim
TGC Tigecycline
CO Colistin
